# Investigation on treatment strategy, prognostic factors, and risk factors for early death in elderly Taiwanese patients with diffuse large B-cell lymphoma

**DOI:** 10.1038/srep44282

**Published:** 2017-03-14

**Authors:** Shih-Feng Cho, Yi-Chang Liu, Hui-Hua Hsiao, Chiung-Tang Huang, Yu-Fen Tsai, Hui-Ching Wang, Sheng-Fung Lin, Ta-Chih Liu

**Affiliations:** 1 Graduate Institute of Clinical Medicine, College of Medicine, Kaohsiung Medical University, Kaohsiung, Taiwan; 2 Division of Haematology and Oncology, Department of Internal Medicine, Kaohsiung Medical University Hospital, Kaohsiung Medical University, Kaohsiung, Taiwan

## Abstract

This study aimed to investigate the treatment strategy, prognostic factors, and risk factors of early death in elderly patients (age ≥ 65 years) with diffuse large B-cell lymphoma (DLBCL) in the rituximab era. Data from elderly patients diagnosed with DLBCL between 2008 and 2014 were collected for analysis. Patients who were younger and had a better performance status were more likely to receive intensive frontline treatment. The median progression-free survival (PFS) and overall survival were 15 and 21 months, respectively. Anthracycline-containing chemotherapy achieved a higher remission rate and showed a trend towards better overall survival but a higher risk of severe neutropenia. Multivariate analysis revealed that very old age (≥81 years), a high-risk age-adjusted international prognostic index (aaIPI) score, and bone marrow involvement were associated with poorer PFS and overall survival. Progression of lymphoma was the major cause of death in the study population. In addition, approximately 25% of patients died within 120 days of being diagnosed. The risk factors for early mortality included very old age, a high-risk aaIPI score, and bone marrow involvement. The appearance of symptoms or signs of tumour lysis syndrome at diagnosis was associated with a trend towards early death.

With increased life expectancy of the overall population, the number of elderly cancer patients is expected to increase in the coming years^1^,^2^,^3^. Regarding lymphoma in particular, a recent study revealed that greater than 50% of new cases of non-Hodgkin’s lymphoma (NHL) occur in patients older than 65 years of age^4^. Indeed, old age is one of the strongest adverse prognostic factors of NHL and is significantly associated with shorter disease-free survival and overall survival^5^,^6^. Furthermore, elderly cancer patients are more likely to suffer from life-threatening chemotherapy-related toxicity or early death due to chronic or weakness-associated diseases and age. It is very important for healthcare professionals to understand the characteristics of this subgroup to ensure that treatment plans are tailored to achieve the best treatment result and to avoid unnecessary side effects.

Diffuse large B-cell lymphoma (DLBCL) is the most common histologic subtype of NHL. In recent decades, the overall survival of patients with DLBCL has improved significantly, the possible causes for which include advances in diagnostic tools, supportive care, and the development and introduction of more potent novel therapeutic agents. For example, the anti-CD20 monoclonal antibody rituximab represented an important milestone in new chemotherapeutic agents for DLBCL treatment. Previous studies have shown that incorporating rituximab into the conventional chemotherapy of cyclophosphamide, doxorubicin, vincristine, and prednisolone (CHOP) significantly improves clinical outcomes, including overall survival and progression-free survival (PFS) in patients with DLBCL^7^,^8^. As a result, the combination of rituximab and conventional chemotherapy has become the standard treatment for DLBCL.

Some studies have evaluated the safety and efficacy of different dosages and chemotherapeutic regimens for treating elderly patients with DLBCL in the rituximab era^9^,^10^,^11^. However, the findings of these studies may not entirely apply to real-world clinical practice, given the strict enrolment and exclusion criteria of clinical trials. In particular, patients with poorer performance and complicated comorbidities are unlikely to have participated in these trials. Another important issue is the occurrence of early death after the diagnosis of cancer or after the start of chemotherapy because elderly patients are more prone to suffer from severe complications of the disease or cancer-related treatment. The risk factors of early death have been evaluated in a previous study, but not in elderly patients with DLBCL^12^.

As the population of elderly cancer patients, including those with DLBCL, is increasing, managing cancer in the elderly has emerged as an increasingly common problem. Accordingly, more studies regarding the treatment strategy and prognostic factors for this patient group are warranted. Hence, we conducted this study to investigate the therapeutic interventions and clinical outcomes of elderly DLBCL patients in real-world clinical practice. In addition, we elucidated the risk factors for early mortality to help physicians tailor treatment plans to achieve optimal clinical outcomes.

## Participants and Methods

### Patient population

We retrospectively analysed patients with DLBCL who were diagnosed at the Kaohsiung Medical University Hospital (KMUH) between 2008 and 2014. This study included elderly patients (aged 65 years or older) with histologically confirmed DLBCL. Patients with primary central nervous system DLBCL were excluded because of the distinct disease characteristics and treatment. The diagnosis and classification were performed according to the 2008 World Health Organization classification of tumours of haematopoietic and lymphoid tissues^13^.

### Data collection

Clinical data were collected by reviewing the medical records of each participant. The data obtained in this study included demographic data, medical history, laboratory data, pathology reports, and imaging studies. Clinical data collection included the following variables: age, gender, lymphoma stage (Ann Arbor stage), Eastern Cooperative Oncology Group (ECOG) performance status (PS), and the presence of B cell-related symptoms. Laboratory data included a complete blood cell count, serum albumin level, serum creatinine level, serum lactate dehydrogenase (LDH) level, beta2-microglobulin level, and hepatitis B/C serology. Bone marrow involvement was determined by pathological review of the bone marrow biopsy. The age-adjusted international prognostic index (aaIPI) was calculated for the analysis^14^, and the Charlson comorbidity index (CCI) was also used to evaluate the impact of comorbidities on clinical outcomes^15^,^16^,^17^.

### Treatment of DLBCL

To treat DLBCL, patients received a combination of rituximab (R) and conventional chemotherapy. The anthracycline-containing (R-CHOP) regimen included rituximab 375 mg/m^2^ on day 1, cyclophosphamide 750 mg/m^2^ on day 2, doxorubicin 50 mg/m^2^ on day 2, vincristine 1.4 mg/m^2^ (up to a maximal dose of 2 mg) on day 2, and prednisolone 40 mg/m^2^ for five days. Doxorubicin was not prescribed for patients receiving R-COP treatment, but the remaining therapeutic agents and schedule were the same as in R-CHOP. The patients were treated every three weeks for six to eight planned treatment courses. If the patients could not tolerate chemotherapy with curative intent, the treatment choices included steroid administration and supportive care. The clinical parameters for treatment planning included age, performance status and comorbidity and were discussed in multi-disciplinary team meetings. The therapeutic intervention for each patient was determined by the physicians and was performed after it was thoroughly explained to the patients and families. Radiotherapy was performed if residual disease was noted after chemotherapy. Autologous peripheral blood stem cell transplantation was conducted for patients with adequate performance status after treatment of relapsed disease. Regarding supportive care, each patient underwent follow-up in haematology outpatient clinics and received laboratory exams, such as complete blood cell counts, on a weekly basis after each course of chemotherapy. For patients found to have severe leukopenia or neutropenia (grade 3 or grade 4), granulocyte-colony stimulating factor (G-CSF) was prescribed immediately. Prophylactic G-CSF was used in the subsequent chemotherapy courses.

### Clinical outcome evaluation

Response to the first-line treatment was evaluated based on International Workshop criteria^18^. The survival analysis included overall survival and PFS. Overall survival was defined as the duration from the date of diagnosis to the date of death from any cause. PFS was defined as the date of diagnosis to the date of lymphoma progression or death from any cause. The patients in this study were strictly followed by maintaining close contact either at home or at the hospital to identify the cause of death, if possible. The causes of death were classified into three main groups: disease progression, treatment-related toxicity, and any other cause. Death caused by treatment-related toxicity was indicated when the mortality was associated with a severe side effect after lymphoma treatment.

To investigate the risk of early mortality, we defined early mortality as death within 120 days of diagnosis^19^.

### Statistics

The frequencies of each categorical variable were compared using chi-square tests (χ2 tests). Logistic regression was performed to analyse the treatment strategy and risk factors for neutropenia. Survival curves were plotted using the Kaplan–Meier method and compared using log-rank test. The hazard ratios (HRs) and 95% confidence intervals (CIs) were calculated using multivariate Cox regression to investigate the relative risks. Additionally, we performed Cox regression in univariate and multivariate analyses to examine the risk factors for early mortality.

All statistical analyses were based on two-sided hypothesis tests with a significance level of P-value < 0.05.

### Ethical approval

This study was conducted according to the Helsinki Declaration. The Institutional Review Board of Kaohsiung Medical University Hospital reviewed and approved the process of acquiring data from the participants (KMUH-IRB-E(I)-20160009).

## Results

### Patient characteristics

There were 145 elderly patients with DLBCL diagnosed between 2008 and 2014. After excluding patients with primary central nervous system DLBCL (n = 9) and incomplete data (n = 3), data from 133 patients (64 male and 69 female) with a median age of 74 years (range 65 to 94 years) were collected in the present study. Detailed information about the study population is listed in Table 1.

### Evaluation of the therapeutic inventions

Of the 133 patients, 23 (17.3%) were receiving steroid monotherapy or supportive care. Additionally, 63 of the patients who received systemic chemotherapy (n = 104) were receiving an R-CHOP regimen. Among patients who were very old (≥81 years) and who had poorer performance scores (ECOG PS 2–4), a significantly higher percentage received less intensive therapy, such as R-COP chemotherapy, steroid monotherapy, or supportive care (Table 2). In addition, there was a trend towards selecting less intensive therapy among patients with a CCI ≥ 1 (see Supplementary Table 1). Multivariate logistic regression revealed that the determining factors for using R-CHOP chemotherapy as initial treatment included a relatively younger age (≤75 years) and good performance status (ECOG PS 0-1) (P < 0.001 and P = 0.012, respectively), but not the absence of comorbidity or a low CCI score (P = 0.303 and P = 0.126, respectively).

The number of cycles of chemotherapy among patients receiving R-CHOP or R-COP chemotherapy ranged from 1 to 8, with average cycles of 5.5 ± 2.2 (R-CHOP: 5.8 ± 1.9 and R-COP: 5.0 ± 2.6). In total, 64 patients (61.5%) completed the planned chemotherapy, including 43 patients (68.3%) in the R-CHOP group and 21 patients (51.2%) in the R-COP group. In the R-CHOP group, 21 (77.8%), 20 (64.5%) and 2 patients (40%) in the 65 to 70, 71 to 80, and greater than 80 years age groups, respectively, completed the planned chemotherapy course. The mean cumulative dose of anthracycline (doxorubicin) was 268.6 mg/m^2^, with an average dose of 45.9 mg/m^2^ per cycle. Additionally, 12 patients (19%) in this group received dose modification (reduction) of anthracycline after the first cycle. The average number of cycles for this subgroup was 5.7 ± 2.1 (range 2 to 8), with a mean cumulative dose of anthracycline of 164.2 mg/m^2^ and an average dose of 28.6 mg/m^2^ per cycle. In the R-COP group, 3 (60%), 15 (62.5%) and 3 patients (25%) among those aged 65 to 70, 71 to 80, and greater than 80 years, respectively, completed the planned chemotherapy course.

Regarding other therapeutic interventions, three patients received radiotherapy for residual lesions and one patient received autologous peripheral blood stem cell transplantation after treatment for relapsed DLBCL.

### Analysis of chemotherapy toxicity

There were 62 patients, including 41 patients in the R-CHOP group (65.1%) and 20 patients in the R-COP group (48.8%), who suffered from grades 3 or 4 leukopenia or neutropenia and received G-CSF administration during first-line chemotherapy treatment. Twenty of the patients receiving G-CSF were hospitalized for neutropenic fever, four of whom died after the infection during hospitalization. Multivariate logistic regression analysis revealed that anthracycline administration was an independent risk factor for grade 3 or 4 leukopenia or neutropenia (P = 0.001). Additionally, three patients received R-CHOP initially and then changed to R-COP due to severe complications (two with neutropenic fever and sepsis and one with acute heart failure).

### Outcome analysis

A total of 62 patients achieved complete remission after first-line treatment, including 42 patients in the R-CHOP group and 20 patients in the R-COP group. After excluding 23 patients who received steroid treatment or supportive care, the overall remission rate was 56.4%. Patients who received R-CHOP chemotherapy had a higher remission rate (66.7%) than those receiving R-COP chemotherapy (48.8%).

**Figure 1:**
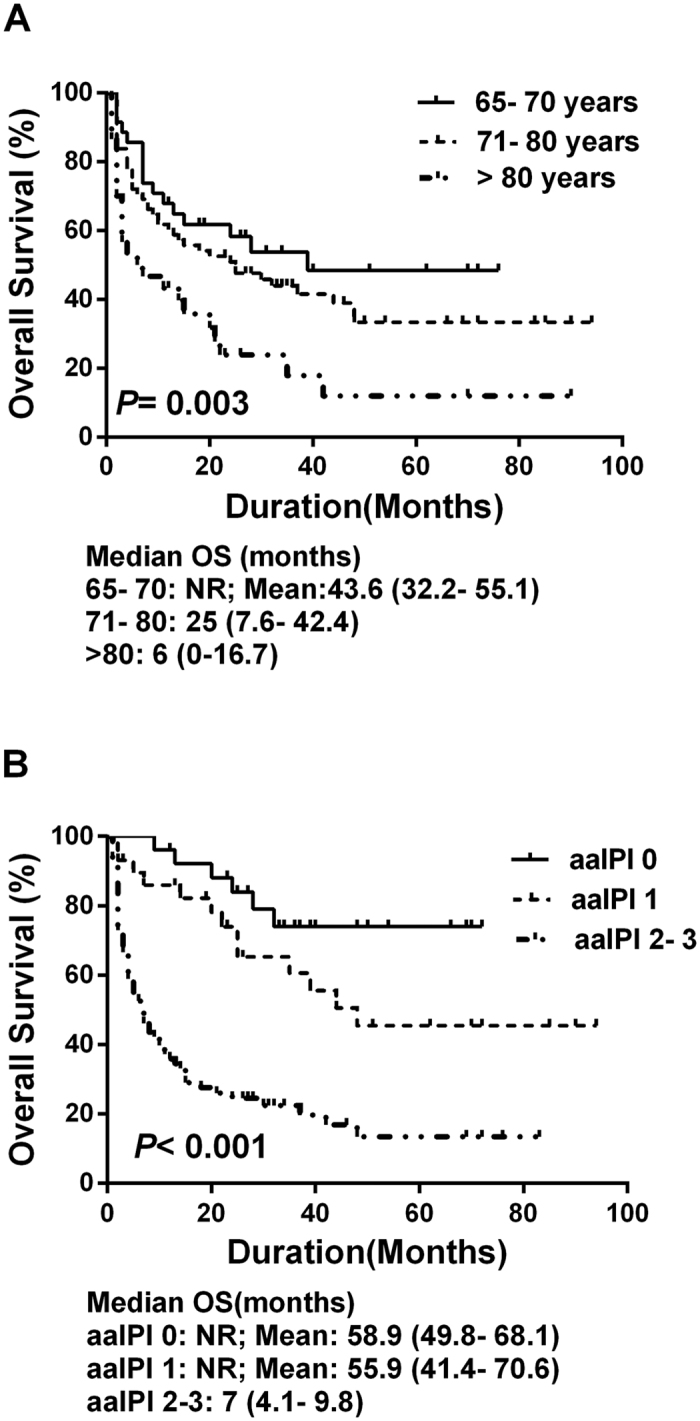
Overall survival of the study population stratified by age (1 A) and aaIPI score (1 B).

The median PFS and overall survival were 15 and 21 months, respectively. Patients with a relatively younger age (65 to 70 years) and a lower aaIPI score exhibited a better prognosis (Fig. 1). With respect to prognostic factors, the multivariate analysis revealed that very old age (≥81 years), a high-risk aaIPI score (2 and 3), and bone marrow involvement were associated with adverse PFS and overall survival (Tables 3 and 4). When patients treated with R-CHOP and R-COP chemotherapy were analysed together, the median PFS and overall survival were 32 and 37 months, respectively. Patients who received R-CHOP chemotherapy showed a trend towards a better median PFS (42 months vs. 15 months, P = 0.067) and overall survival (44 months vs. 25 months, P = 0.080) than patients in the R-COP group. Multivariate analysis revealed that a high-risk aaIPI score, bone marrow involvement and an abnormal beta2-microglobulin level showed a trend for poorer PFS (see Supplementary Table 2). A high-risk aaIPI score was an independent adverse prognostic factor for overall survival (see Supplementary Table 3).

A total of 81 patients died during follow-up, including 44 patients who died from lymphoma progression. No patients died of acute heart failure after chemotherapy. The detailed causes of death, including early and later mortality, are presented in Table 5.

### Identification of risk factors for early mortality

Thirty-four patients (25.6%) with early mortality (i.e., those who died within 120 days of diagnosis) were identified. Of these 34 patients, 5, 15, and 14 were aged 65 to 70, 71 to 80, and greater than 80 years, respectively (see Supplementary Table 4). The causes of early mortality included rapid progression of lymphoma (*n* = 24, 70.6%), treatment-related toxicity (*n* = 4, 11.8%), and organ failure as a result of other causes (*n* = 6, 17.6%). Multivariate Cox regression analysis revealed that very old age (≥81 years), a high-risk aaIPI score (2 to 3), and bone marrow involvement were independent risk factors. Patients who had symptoms or signs of tumour lysis syndrome at diagnosis showed a trend towards early mortality (Table 6).

## Discussion

In this retrospective study, we evaluated the treatment strategies, clinical outcomes, and risk factors for early mortality in patients with DLBCL. The results revealed that the most important factors determining initial treatment were patients’ age and performance status. We found that physicians significantly reduced their usage of anthracycline in chemotherapy regimens for older patients and those with poorer performance status. Approximately 35% of patients did not receive anthracycline-containing chemotherapy, and this proportion was consistent with the findings of previous studies^20^,^21^. Possible explanations for this lower usage included concerns about the major side effects of anthracycline, such as the higher rate of cardiotoxicity and myelosuppression in this population^22^. A small portion of patients with no comorbidity, low CCI (CCI = 0) or relatively good performance status (ECOG 0-1) received steroid monotherapy or supportive care. Most of these patients were older, were in an advanced stage or had a high-risk aaIPI score. Based on this finding, age and disease status might be objective factors that determine treatment choice. Patient preference for palliation may also affect the treatment strategy. Given the retrospective nature of the present study, recommendations regarding the treatment choice for these elderly patients remain hypothesis generating only, and additional prospective studies are warranted to address this issue.

This study also investigated prognostic factors. Some findings were concordant with the results of previous studies, whereas others were not. The independent prognostic factors identified in this study included a high-risk aaIPI score, very old age and bone marrow involvement, which were also noted in previous studies^14^,^16^,^23^. In the present study, patients with very old age (≥81 years) showed a reduced median overall survival compared with the survival reported in a previous study^17^. One possible explanation for this difference is the higher percentage (30%) of patients receiving supportive care or steroid monotherapy in our study, which indicated that certain patients who could tolerate treatment with curative intent were undertreated. Additionally, the present study showed that the presence of comorbidities was not associated with poor prognosis, and this finding was consistent with the results of a previous study^17^. If the data were analysed based on the CCI, there might have been a trend towards poorer long-term outcomes.

This study revealed that patients who received more intensive treatment, such as R-CHOP chemotherapy, had a higher rate of complete remission, which may translate to better long-term prognosis. However, anthracycline administration was associated with a high risk of side effects. Our study showed that approximately 65% of patients receiving R-CHOP chemotherapy required G-CSF administration for severe neutropenia. This finding emphasized the importance of supportive care and pretreatment evaluation. In our study, primary prophylaxis with G-CSF administration was not routinely performed. To reduce the risk of neutropenic fever, closely monitoring blood cell counts and using G-CSF for secondary prevention might be an alternative choice. The results of the present study also suggested that substantial effort should be made to identify patients who can tolerate intensive therapy with a curative intent and to select patients who may benefit from less intensive treatment to provide meaningful quality and quantity of life. Several models, including functional status evaluations and comorbidity indices, have been used or are under development, such as the cumulative illness rating scale, CCI, and comprehensive geriatric assessment^24^,^25^. With more comprehensive evaluations of different aspects of patient’s health, such as comorbidities, as well as functional, social, and psychological status, physicians will have more information to better tailor the corresponding treatment strategies^26^.

The causes and risk factors of early mortality were also investigated in this study. Approximately 25% of patients died within 120 days of their diagnosis despite the advances in supportive care and the use of a novel agent. The most common causes of early death were progression of lymphoma and severe infection due to treatment-related neutropenia. This finding suggested that disease status and treatment may play important roles, which was consistent with the results of previous studies^27^,^28^. Regarding the risk factors for early mortality, our findings indicated that very old age (≥81 years), a high-risk aaIPI score, and bone marrow involvement by lymphoma were risk factors. Additionally, patients with symptoms or signs of tumour lysis syndrome at diagnosis showed a trend towards early death. The appearance of the previously mentioned factors may indicate a more complicated clinical situation, increased tumour burden, or advanced disease status; therefore, physicians should also pay close attention to this subgroup of patients.

In conclusion, the present study provided information about the treatment strategies, chemotherapy toxicity, long-term prognostic factors, and risk factors of early mortality in DLBCL. Treatment of elderly patients with DLBCL remains a challenge in clinical practice, and comprehensive evaluations to tailor therapeutic interventions and offer the best supportive care may reduce complications and improve these patients’ clinical outcomes.

## Additional Information

**How to cite this article**: Cho, S.-F. *et al*. Investigation of treatment strategy, prognostic factors, and risk factors for early death in elderly patients with diffuse large B-cell lymphoma. *Sci. Rep.* **7**, 44282; doi: 10.1038/srep44282 (2017).

**Publisher's note:** Springer Nature remains neutral with regard to jurisdictional claims in published maps and institutional affiliations.

## Supplementary Material

Supplementary Information

## Figures and Tables

**Table 1:** General characteristics of the elderly DLBCL patients.

Total patients*, n* = 133	*n* (%)
Median age (±SD)	74 (6.6)
Age group (years)	
65–70	35 (26.3)
71–80	68 (51.1)
≥81	30 (22.6)
Gender	
Male	64 (51.9)
Female	69 (48.1)
ECOG PS	
0–1	98 (73.7)
2–4	35 (26.3)
Ann Arbor stage	
I-II	50 (37.6)
III-IV	83 (62.4)
aaIPI	
0	25 (18.8)
1	30 (22.6)
2–3	78 (58.6)
Extranodal involvement	80 (60.2)
Bone marrow involvement	23 (17.3)
Presence of B symptoms	38 (28.6)
Abnormal LDH level	93 (69.9)
Anaemia (Haemoglobin <12 g/dl)	73 (54.9)
Abnormal B2M level	58 (43.6)
Low albumin level (<3.5 g/dl)	66 (49.6)
Renal function impairment	43 (32.3)
Diabetes mellitus	33 (24.8)
HBV	24 (18.0)
HCV	20 (15.0)
With comorbidity	98 (73.7)
CCI ≥ 1	62 (46.6)
Symptoms of TLS at diagnosis	9 (6.8)

The number of patients in each Ann Arbor stage: 1:18; 2:32; 3:34; 4:49.

The number of patients in each ECOG PS: 0:14; 1:84; 2:17; 3:10; 4:8.

The number of patients with different aaIPI scores: 0:25; 1:30; 2:47; 3:31.

aaIPI, age-adjusted international prognostic index; B2M, beta2-microglobulin; CCI, Charlson comorbidity index; DLBCL, diffuse large B-cell lymphoma; ECOG PS, Eastern Cooperative Oncology Group Performance Status; HBV, hepatitis B virus; HCV, hepatitis C virus; LDH, lactate dehydrogenase; TLS, tumour lysis syndrome.

**Table 2:** Distribution of initial treatments in the study population stratified by age and performance.

Treatment choices	Age group*			ECOG PS group^+^	
	65–70 (*n* = 35)	71–80 (*n* = 68)	≥81 (*n* = 30)	0–1 (*n* = 98)	2–4 (*n* = 35)
No treatment or steroid monotherapy (*n*, %)	2 (5.7)	12 (17.6)	9 (30)	8 (8.2)	15 (42.9)
R-CHOP (*n*, %)	27 (77.1)	31 (45.6)	5 (16.7)	56 (57.1)	7 (20)
R-COP (*n*, %)	5 (14.3)	24 (35.3)	12 (40)	32 (32.7)	9 (25.7)
Other regimens (*n*, %)	1 (2.9)	1 (1.5)	4 (13.3)	2 (2.0)	4 (11.4)

*P-value < 0.001.

^+^*P*-value < 0.001.

Other regimens included the following combinations: rituximab, vincristine, and prednisolone; rituximab and prednisolone. ECOG PS, Eastern Cooperative Oncology Group Performance Status; R, rituximab; CHOP, cyclophosphamide, doxorubicin, vincristine, and prednisolone; COP, cyclophosphamide, vincristine, and prednisolone.

**Table 3:** The analysis of prognostic factors of progression-free survival by univariate and multivariate Cox regression.

Variables	Univariate analysis		Multivariate analysis*	
	HR 95% CI	*P*-value	HR 95% CI	*P*-value
Age (≥81 years)	2.18 (1.34–3.54)	0.002	2.46 (1.28–4.74)	0.007
Male gender	1.03 (0.67–1.58)	0.886		
Performance status 2–4	2.59 (1.65–4.08)	<0.001	1.27 (0.69–2.29)	0.438
aaIPI 2 or 3	4.32 (2.61–6.02)	<0.001	2.75 (1.16–6.48)	0.021
BM involvement	2.37 (1.43–3.94)	0.001	2.28 (1.27–4.12)	0.006
Stage 3–4	3.59 (2.14–6.02)	<0.001	1.34 (0.62–2.91)	0.452
With B symptoms	1.50 (0.96–2.36)	0.078	1.10 (0.66–1.84)	0.704
Abnormal LDH level	2.42 (1.43–4.08)	0.001	0.94 (0.48–1.84)	0.850
Abnormal B2M level	2.38 (1.55–3.68)	<0.001	1.41 (0.83–2.39)	0.202
Low albumin level	2.06 (1.33–3.18)	0.001	1.15 (0.69–1.93)	0.594
Renal function impairment	1.63 (1.04–2.04)	0.031	0.84 (0.49–1.49)	0.516
Symptoms of TLS at diagnosis	3.60 (1.70–7.61)	0.001	2.19 (0.81–5.92)	0.122
At least 1 comorbidity	1.13 (0.69–1.83)	0.634		
CCI ≥ 1	1.35 (0.88–2.06)	0.172		

^*^Factors with a P-value less than 0.1 in the univariate analysis were entered into the multivariate analysis.

aaIPI, age-adjusted international prognostic index; B2M, beta2-microglobulin; CCI, Charlson comorbidity index; HR, hazard ratio; LDH, lactate dehydrogenase; TLS, tumour lysis syndrome.

**Table 4:** Univariate and multivariate Cox regression analysis of prognostic factors of overall survival.

Variables	Univariate analysis		Multivariate analysis*	
	HR 95%CI	*P*-value	HR 95%CI	*P*-value
Age (≥81 years)	2.52 (1.54–4.13)	<0.001	2.69 (1.39–5.23)	0.003
Male gender	0.94 (0.61–1.46)	0.788		
Performance status 2–4	4.36 (2.49–7.65)	<0.001	1.27 (0.69–2.31)	0.433
aaIPI 2 or 3	4.49 (2.66–7.60)	<0.001	3.08 (1.26–7.49)	0.013
BM involvement	2.02 (1.21–3.39)	0.008	1.97 (1.07–3.61)	0.029
Stage 3–4	3.40 (2.01–5.78)	<0.001	1.17 (0.53–2.58)	0.707
With B symptoms	1.54 (0.97–2.44)	0.069	1.09 (0.65–1.84)	0.745
Abnormal LDH level	2.52 (1.46–4.37)	0.001	0.97 (0.48–1.94)	0.922
Abnormal B2M level	2.49 (1.59–3.89)	<0.001	1.49 (0.87–2.58)	0.145
Low albumin level	2.23 (1.42–3.48)	<0.001	1.26 (0.74–2.15)	0.402
Renal function impairment	1.72 (1.09–2.71)	0.019	0.85 (0.49–1.48)	0.562
Symptoms of TLS at diagnosis	3.69 (1.74–7.83)	0.001	2.22 (0.82–6.03)	0.118
At least 1 comorbidity	1.21 (0.73–2.01)	0.456		
CCI ≥ 1	1.42 (0.92–2.20)	0.115		

^*^Factors with P-value less than 0.1 in the univariate analysis were entered into the multivariate analysis. aaIPI, age-adjusted international prognostic index; B2M, beta2-microglobulin; CCI, Charlson comorbidity index; HR, hazard ratio; LDH, lactate dehydrogenase; TLS, tumour lysis syndrome.

**Table 5:** Classification of causes of death based on therapeutic intervention.

Treatment choices	Mortality (*n*)	Main causes of death^a^		
		Lymphoma (*n*)	Toxicity (*n*)	Other (*n*)^b^
No treatment or steroid only (23)	23	21	0	2
R-CHOP (63)	28	11	2	15
R-COP (41)	25	9	3	13
Other regimens (6)	5	3	1	1

^a^The causes of death included early and late mortality.

^b^Respiratory failure, cardiac failure and shock due to gastrointestinal bleeding. R, rituximab; CHOP, cyclophosphamide, doxorubicin, vincristine, and prednisolone; COP, cyclophosphamide, vincristine, and prednisolone.

**Table 6:** Investigation of the risk factors of early mortality using univariate and multivariate Cox regression analysis.

Variables	Univariate analysis		Multivariate analysis*	
	HR 95% CI	*P*-value	HR 95% CI	*P*-value
Very old age (≥81 years)	3.16 (1.59–6.23)	0.001	4.69 (1.79–12.27)	0.002
Male gender	1.23 (0.63–2.42)	0.541		
Performance status 2–4	3.21 (1.64–6.31)	0.001	1.06 (0.47–2.40)	0.880
aaIPI 2 or 3	13.15 (3.15–54.93)	<0.001	7.43 (1.08–51.09)	0.041
BM involvement	2.58 (1.26–5.29)	0.010	2.74 (1.13–6.61)	0.025
Stage 3 or 4	4.97 (1.75–14.12)	0.003	0.69 (0.17–2.79)	0.602
Abnormal LDH level	7.59 (1.82–31.72)	0.005	1.49 (0.29–7.62)	0.635
Abnormal B2M level	2.64 (1.30–5.33)	0.007	1.05 (0.45–2.42)	0.915
Low albumin level	3.17 (1.48–6.80)	0.003	1.38 (0.58–3.83)	0.464
Renal function impairment	2.40 (1.22–4.71)	0.011	1.00 (0.42–2.36)	0.998
Presence with B symptom	2.56 (1.31–5.03)	0.006	1.67 (0.76–3.67)	0.204
Symptoms of TLS at diagnosis	4.26 (1.75–10.36)	0.001	2.96 (0.86–10.19)	0.086
At least 1 comorbidity	2.24 (0.87–5.78)	0.096	1.63 (0.59–4.47)	0.342
CCI ≥ 1	1.29 (0.66–2.55)	0.448		

^*^Factors with P-value less than 0.1 in the univariate analysis were entered into the multivariate logistic regression model. aaIPI, age-adjusted international prognostic index; B2M, beta2-microglobulin; CCI, Charlson comorbidity index; HR, hazard ratio; LDH, lactate dehydrogenase; TLS, tumour lysis syndrome.

## References

[b1] Campisi J. Aging, cellular senescence, and cancer. Annual review of physiology 75, 685–705, doi: 10.1146/annurev-physiol-030212-183653 (2013). PMC4166529 23140366

[b2] Falandry C., Bonnefoy M., Freyer G. & Gilson E. Biology of cancer and aging: a complex association with cellular senescence. Journal of clinical oncology: official journal of the American Society of Clinical Oncology 32, 2604–2610, doi: 10.1200/JCO.2014.55.1432 (2014). 25071126

[b3] Hoeijmakers J. H. DNA damage, aging, and cancer. The New England journal of medicine 361, 1475–1485, doi: 10.1056/NEJMra0804615 (2009). 19812404

[b4] Balducci L. & Extermann M. Cancer and aging. An evolving panorama. Hematology/oncology clinics of North America 14, 1–16 (2000). 10680068 10.1016/s0889-8588(05)70274-4

[b5] Dixon D. O. . Effect of age on therapeutic outcome in advanced diffuse histiocytic lymphoma: the Southwest Oncology Group experience. Journal of clinical oncology: official journal of the American Society of Clinical Oncology 4, 295–305 (1986). 3512783 10.1200/JCO.1986.4.3.295

[b6] Vose J. M. . The importance of age in survival of patients treated with chemotherapy for aggressive non-Hodgkin’s lymphoma. Journal of clinical oncology: official journal of the American Society of Clinical Oncology 6, 1838–1844 (1988). 2462026 10.1200/JCO.1988.6.12.1838

[b7] Coiffier B. . CHOP chemotherapy plus rituximab compared with CHOP alone in elderly patients with diffuse large-B-cell lymphoma. The New England journal of medicine 346, 235–242, doi: 10.1056/NEJMoa011795 (2002). 11807147

[b8] Pfreundschuh M. . CHOP-like chemotherapy plus rituximab versus CHOP-like chemotherapy alone in young patients with good-prognosis diffuse large-B-cell lymphoma: a randomised controlled trial by the MabThera International Trial (MInT) Group. The lancet oncology 7, 379–391, doi: 10.1016/S1470-2045(06)70664-7 (2006). 16648042

[b9] Musolino A. . Activity and safety of dose-adjusted infusional cyclophosphamide, doxorubicin, vincristine, and prednisone chemotherapy with rituximab in very elderly patients with poor-prognostic untreated diffuse large B-cell non-Hodgkin lymphoma. Cancer 117, 964–973, doi: 10.1002/cncr.25582 (2011). 20960528

[b10] Meguro A. . Rituximab plus 70% cyclophosphamide, doxorubicin, vincristine and prednisone for Japanese patients with diffuse large B-cell lymphoma aged 70 years and older. Leukemia & lymphoma 53, 43–49, doi: 10.3109/10428194.2011.600486 (2012). 21864040

[b11] Merli F. . Cyclophosphamide, doxorubicin, vincristine, prednisone and rituximab versus epirubicin, cyclophosphamide, vinblastine, prednisone and rituximab for the initial treatment of elderly “fit” patients with diffuse large B-cell lymphoma: results from the ANZINTER3 trial of the Intergruppo Italiano Linfomi. Leukemia & lymphoma 53, 581–588, doi: 10.3109/10428194.2011.621565 (2012). 21895543

[b12] Soubeyran P. . Predictors of early death risk in older patients treated with first-line chemotherapy for cancer. Journal of clinical oncology: official journal of the American Society of Clinical Oncology 30, 1829–1834, doi: 10.1200/JCO.2011.35.7442 (2012). 22508806

[b13] Vardiman J. W. . The 2008 revision of the World Health Organization (WHO) classification of myeloid neoplasms and acute leukemia: rationale and important changes. Blood 114, 937–951, doi: 10.1182/blood-2009-03-209262 (2009). 19357394

[b14] A predictive model for aggressive non-Hodgkin’s lymphoma. The International Non-Hodgkin’s Lymphoma Prognostic Factors Project. The New England journal of medicine 329, 987–994, doi: 10.1056/NEJM199309303291402 (1993). 8141877

[b15] Charlson M. E., Pompei P., Ales K. L. & MacKenzie C. R. A new method of classifying prognostic comorbidity in longitudinal studies: development and validation. Journal of chronic diseases 40, 373–383 (1987). 3558716 10.1016/0021-9681(87)90171-8

[b16] Lin T. L. . The impact of age, Charlson comorbidity index, and performance status on treatment of elderly patients with diffuse large B cell lymphoma. Annals of hematology 91, 1383–1391, doi: 10.1007/s00277-012-1463-9 (2012). 22526364

[b17] Thieblemont C. . Non-Hodgkin’s lymphoma in very elderly patients over 80 years. A descriptive analysis of clinical presentation and outcome. Annals of oncology: official journal of the European Society for Medical Oncology/ESMO 19, 774–779, doi: 10.1093/annonc/mdm563 (2008). 18065404

[b18] Cheson B. D. . Report of an international workshop to standardize response criteria for non-Hodgkin’s lymphomas. NCI Sponsored International Working Group. Journal of clinical oncology: official journal of the American Society of Clinical Oncology 17, 1244 (1999). 10561185 10.1200/JCO.1999.17.4.1244

[b19] Bairey O., Bar-Natan M. & Shpilberg O. Early death in patients diagnosed with non-Hodgkin’s lymphoma. Annals of hematology 92, 345–350, doi: 10.1007/s00277-012-1623-y (2013). 23161388

[b20] Maartense E. . Elderly patients with non-Hodgkin’s lymphoma: population-based results in The Netherlands. Annals of oncology: official journal of the European Society for Medical Oncology/ESMO 9, 1219–1227 (1998). 10.1023/a:1008485722472 9862053

[b21] Grann V. R. . Outcomes and diffusion of doxorubicin-based chemotherapy among elderly patients with aggressive non-Hodgkin lymphoma. Cancer 107, 1530–1541, doi: 10.1002/cncr.22188 (2006). 16933332

[b22] Swain S. M., Whaley F. S. & Ewer M. S. Congestive heart failure in patients treated with doxorubicin: a retrospective analysis of three trials. Cancer 97, 2869–2879, doi: 10.1002/cncr.11407 (2003). 12767102

[b23] Campbell J. . The prognostic impact of bone marrow involvement in patients with diffuse large cell lymphoma varies according to the degree of infiltration and presence of discordant marrow involvement. European journal of haematology 76, 473–480, doi: 10.1111/j.1600-0609.2006.00644.x (2006). 16529599

[b24] Parmelee P. A., Thuras P. D., Katz I. R. & Lawton M. P. Validation of the Cumulative Illness Rating Scale in a geriatric residential population. Journal of the American Geriatrics Society 43, 130–137 (1995). 7836636 10.1111/j.1532-5415.1995.tb06377.x

[b25] Extermann M. & Hurria A. Comprehensive geriatric assessment for older patients with cancer. Journal of clinical oncology: official journal of the American Society of Clinical Oncology 25, 1824–1831, doi: 10.1200/JCO.2007.10.6559 (2007). 17488980

[b26] Morrison V. A. . Diffuse large B-cell lymphoma in the elderly: impact of prognosis, comorbidities, geriatric assessment, and supportive care on clinical practice. An International Society of Geriatric Oncology (SIOG) expert position paper. Journal of geriatric oncology 6, 141–152, doi: 10.1016/j.jgo.2014.11.004 (2015). 25491101

[b27] Gomez H. . Risk factors for treatment-related death in elderly patients with aggressive non-Hodgkin’s lymphoma: results of a multivariate analysis. Journal of clinical oncology: official journal of the American Society of Clinical Oncology 16, 2065–2069 (1998). 9626205 10.1200/JCO.1998.16.6.2065

[b28] Dumontet C. . Factors predictive of early death in patients receiving high-dose CHOP (ACVB regimen) for aggressive non-Hodgkin’s lymphoma: a GELA study. British journal of haematology 118, 210–217 (2002). 12100150 10.1046/j.1365-2141.2002.03565.x

