# Supplementary material for: Investigation on treatment strategy, prognostic factors, and risk factors for early death in elderly Taiwanese patients with diffuse large B-cell lymphoma
Source: Sci Rep. 2017 Mar 14;7:44282. doi: 10.1038/srep44282 (PMC5349600; doi:10.1038/srep44282)
Supplement: Supplementary Information [file srep44282-s1.pdf]

Supplementary Information

Investigation on treatment strategy, prognostic factors, and risk factors for early death in elderly Taiwanese patients with diffuse large B-cell lymphoma

Shih-Feng Cho[1, 2], Yi-Chang Liu[2], Hui-Hua Hsiao[2], Chiung-Tang Huang[2], Yu-Fen Tsai[2], Hui-Ching Wang[2], Sheng-Fung Lin[2], Ta-Chih Liu[1, 2]*

1. Graduate Institute of Clinical Medicine, College of Medicine, Kaohsiung Medical University, Kaohsiung, Taiwan

2. Division of Haematology and Oncology, Department of Internal Medicine, Kaohsiung Medical University Hospital, Kaohsiung Medical University, Kaohsiung, Taiwan

Correspondence to: Ta-Chih Liu

Affiliation: Division of Haematology and Oncology, Department of Internal Medicine, Kaohsiung Medical University Hospital, Kaohsiung Medical University, Kaohsiung, Taiwan

Address: No.100, Tzyou 1st Road Kaohsiung 807, Taiwan

Email: d730093@cc.kmu.edu.tw

Tel: +886-7-312-1101 ext. 6110

Fax: +886-7-316-2429

SUPPLEMENTARY TABLES

Supplementary Table 1. Distribution of initial treatments in study population stratified by Charlson comorbidity index (CCI) and the presence of comorbidity

	Classified by CCI*		Classified by comorbidity[+]	
Treatment choices	CCI= 0 (*n*= 71)	CCI ≥1 (*n*= 62)	Without (*n*= 35)	With (*n*= 98)
No treatment or steroid monotherapy (*n*, %)	10 (14.1)	13 (20.9)	3 (8.6)	20 (20.4)
R-CHOP (*n*, %)	40 (56.3)	23 (37.1)	22 (62.8)	41 (41.8)
R-COP (*n*, %)	20 (28.2)	21 (33.9)	9 (25.7)	32 (32.7)
Other regimens (*n*, %)	1 (1.4)	5 (8.1)	1 (2.9)	5 (5.1)

**P*-value = 0.061

[+]*P*-value = 0.144

Other regimens included the following combination: rituximab, vincristine and prednisolone; rituximab and prednisolone

Supplementary Table 2. The analysis for prognostic factors of progression-free survival by univariate and multivariable Cox regression in patients received R-CHOP or R-COP chemotherapy.

**Variables**	**Univariate analysis**		**Multivariate analysis***	
	**HR 95% CI**	***P*-value**	**HR 95% CI**	***P*-value**
Age (≥81 years)	1.62 (0.81-3.21)	0.171		
Male gender	0.85 (0.50-1.43)	0.538		
Performance status 2-4	1.64 (0.85-3.18)	0.141		
aaIPI 2 or 3	3.86 (2.16-6.89)	<0.001	2.22 (0.92-5.39)	0.078
BM involvement	2.66 (1.42-4.98)	0.002	1.86 (0.92-3.76)	0.082
Stage 3-4	3.89 (2.08-7.29)	<0.001	1.86 (0.77-4.50)	0.167
With B symptoms	1.23 (0.69-2.19)	0.485		
Abnormal LDH level	2.28 (1.22-4.24)	0.010	0.95 (0.43-2.07)	0.893
Abnormal B2M level	2.14 (1.26-3.63)	0.005	1.73 (0.95-3.17)	0.073
Low albumin level	1.58 (0.93-2.67)	0.088	1.01 (0.55-1.85)	0.965
Renal function impairment	1.13 (0.62-2.04)	0.693		
Symptoms of TLS at diagnosis	3.15 (0.97-10.21)	0.056	1.36 (0.40-4.60)	0.621
At least 1 comorbidity	0.80 (0.46-1.41)	0.441		
CCI ≥1	1.13 (0.67-1.91)	0.646		

* Factors with P-value less than 0.1 in the univariate analysis were entered into the multivariate logistic regression model.

aaIPI, age-adjusted international prognostic index; B2M, beta2-microglobulin; CCI, Charlson comorbidity index; HR, hazard ratio; LDH lactate dehydrogenase; TLS, tumour lysis syndrome.

Supplementary Table 3. The analysis for prognostic factors of overall survival by univariate and multivariable Cox regression in patients received R-CHOP or R-COP chemotherapy.

**Variables**	**Univariate analysis**		**Multivariate analysis***	
	**HR 95% CI**	***P*-value**	**HR 95% CI**	***P*-value**
Age (≥ 81 years)	2.02 (1.00-4.05)	0.049	2.15 (0.94-4.93)	0.071
Male gender	0.73 (0.42-1.27)	0.260		
Performance status 2-4	4.89 (1.99-12.01)	0.001	0.91 (0.39-2.08)	0.826
aaIPI 2 or 3	2.45 (1.62-3.71)	<0.001	2.87 (1.05-7.84)	0.040
BM involvement	2.08 (1.09-3.97)	0.026	1.65 (0.77-3.52)	0.197
Stage 3-4	3.42 (1.82-6.41)	<0.001	1.46 (0.58-3.67)	0.419
With B symptoms	1.23 (0.68-2.24)	0.498		
Abnormal LDH level	2.25 (1.18-4.29)	0.013	0.90 (0.39-2.12)	0.815
Abnormal B2M level	2.17 (1.26-3.76)	0.005	1.56 (0.80-3.06)	0.191
Low albumin level	1.69 (0.98-2.89)	0.058	1.05 (0.55-2.01)	0.883
Renal function impairment	1.17 (0.64-2.17)	0.609		
Symptoms of TLS at diagnosis	3.39 (1.04-11.04)	0.042	1.82 (0.45-7.37)	0.403
At least 1 comorbidity	0.83 (0.46-1.47)	0.518		
CCI ≥ 1	1.17 (0.68-2.01)	0.570		

* Factors with P-value less than 0.1 in the univariate analysis were entered into the multivariate logistic regression model.

aaIPI, age-adjusted international prognostic index; B2M, beta2-microglobulin; CCI, Charlson comorbidity index; HR, hazard ratio; LDH lactate dehydrogenase; TLS, tumour lysis syndrome.

Supplementary Table 4. The characteristics of the patients with or without early mortality.

Total patients, *n* = 133	Early mortality(+) *n*= 34	Early mortality(-) *n*= 99	*P*-value
Male gender (*n*, %)	18 (52.9)	46 (46.5)	0.555
Median age (± SD)	78 (6.9)	73 (6.3)	0.404
Age group (years)			0.007
65-70 (*n*, %)	5 (14.7)	30 (30.3)	
71-80 (*n*, %)	15 (44.1)	53 (53.5)	
≥ 81 (*n*, %)	14 (41.2)	16 (16.2)	
ECOG PS			0.001
0-1 (*n*, %)	17 (50)	81 (81.8)	
2-4 (*n*, %)	17 (50)	18 (18.2)	
Ann Arbor stage			<0.001
I-II (*n*, %)	4 (11.8)	46 (46.5)	
III-IV (*n*, %)	30 (88.2)	53 (53.5)	
aaIPI			<0.001
0 (*n*, %)	0	25 (25.2)	
1 (*n*, %)	2 (5.9)	28 (28.3)	
2-3 (*n*, %)	32 (94.1)	46 (46.5)	
Bone marrow involvement (*n*, %)	11 (32.4)	12 (12.1)	0.016
Extranodal involvement (*n*, %)	18 (52.9)	62 (62.6)	0.417
Presence of B symptom (*n*, %)	16 (47.1)	22 (22.2)	0.008
Presence of symptom of TLS (*n*, %)	6 (17.6)	3 (3.3)	0.009
Abnormal LDH level (*n*, %)	32 (94.1)	61 (61.6)	<0.001
Anaemia (*n*, %)	22 (64.7)	51 (51.5)	0.232
Abnormal B2M level (*n*, %)	22 (64.7)	36 (36.4)	0.005
Low albumin level (*n*, %)	25 (73.5)	41 (41.4)	0.001
Renal function impairment (*n*, %)	17 (50)	26 (26.3)	0.019
With comorbidity (*n*, %)	29 (85.3)	69 (69.7)	0.113
CCI≥1(*n*, %)	18 (52.9)	44 (44.4)	0.392

aaIPI, age-adjusted international prognostic index; B2M, beta2-microglobulin; CCI, Charlson comorbidity index; ECOG PS, Eastern Cooperative Oncology Group Performance Status; LDH, lactate dehydrogenase; TLS, tumour lysis syndrome.
